# Frailty in people living with HIV

**DOI:** 10.1186/s12981-018-0210-2

**Published:** 2018-11-16

**Authors:** Mark Bloch

**Affiliations:** Holdsworth House Medical Practice, Darlinghurst, Level 3, 26 College St, Sydney, NSW 2010 Australia

**Keywords:** HIV, Frailty, Ageing, Comorbidities

## Abstract

The life expectancy of people living with HIV (PLHIV) has dramatically improved with effective and well-tolerated antiretroviral therapy. This presents a new challenge in caring for this patient population, with up to 28% of older PLHIV being identified as frail. Studies suggest that the prevalence of frailty is higher in PLHIV compared to the general population, and that the onset of frailty occurs at an earlier age. Frail individuals often present with multiple and non-specific health complaints, fluctuating disability, falls and delirium, and are at higher risk for multiple adverse outcomes, post-operative complications, poor responses to vaccination and functional decline. They tend to require longer hospital admissions, are more likely to require nursing home care, and are at greater risk of mortality. The degree of frailty can fluctuate over time. Limited evidence exists to support the reversal of frailty, but epidemiological evidence suggests that interventions to assess and manage co-morbidities, reducing risk factors such as smoking, increasing exercise and optimising BMI, and improving personal and community resources, are all likely to reduce the risk of frailty. Physicians who care for PLHIV need to recognise and manage frailty in this patient population. This includes an understanding of: when to intervene aggressively in the management of an older patient with a new HIV diagnosis to delay or prevent permanent debility and frailty; when to acknowledge that the patient has become frail; and the role of geriatric medicine in addressing the specific issues and needs of this patient, such as maximising functional ability, preventing falls, reducing social isolation and improving quality of life.

## Background

Due to the success of antiretroviral therapy (ART) in extending the lifespan of people living with HIV (PLHIV), and the reduction in incident HIV infections, the cohort of PLHIV has been gradually ageing and the median age is approaching 50 in most Western clinics [1]. As a result, frailty has increasingly become an issue of concern for those who manage patients with HIV.

Increasing evidence suggests that whilst PLHIV do not age prematurely, concerns regarding frailty normally seen in a geriatric population can often be observed in patients with HIV a decade or more earlier [2, 3]. In addition, the prevalence of comorbidities, multi-morbidity and frailty is higher in PLHIV than the general population at all ages, and the gap between the two populations widens with age [4–6].

Several factors predispose PLHIV to higher rates of frailty, including the effects of chronic inflammation from HIV (even with complete plasma virological suppression), the toxic effects of earlier antiretroviral regimens, delayed initiation of therapy for HIV, higher rates of multi-morbidity (the presence of two or more chronic medical conditions) and co-infection, HIV-associated neurocognitive disorder, lifestyle factors such as smoking, and poverty and social isolation [7].

## Definition of frailty

There is redundancy in living systems that allows for loss of function that can be compensated for, which can be measured by deficit accumulation. Accumulation of deficits over time predisposes an individual to the development of frailty [8]. Frailty has been defined as an increased vulnerability to stressors due to a lack of reserve and reduced ability to compensate for disruptions to homeostasis [8]. Frailty demonstrates that biological age may vary at the same chronological age.

There are 3 different ways of defining frailty:

1. As a clinical syndrome or phenotype, defined by Fried et al. [9].
2. Through the subjective opinion of the clinician.
3. Using multidimensional risk state or frailty index (FI) [10, 11].

### Fried phenotype of frailty

The Fried phenotype of frailty is defined as three or more of five parameters: [9].

1. Unintentional weight loss of more than 10 lbs in the previous year.
2. Self-reported exhaustion.
3. Weak grip strength.
4. Slow walking speed.
5. Reduced physical activity.

Patients with one or two of these characteristics are classified as ‘pre-frail’ [9].

The Fried definition of frailty has clinical coherency and reproducibility, and includes sarcopenia and its pathophysiological manifestations in its characterisation of frailty. However, it doesn’t account for neurocognitive and psychosocial aspects of frailty and relies on performance-based tests [9].

### Subjective opinion

Subjective assessment of biological age and frailty that considers appearance, mobility and communication can have a good inter-rater correlation [12]. A seven-stage clinical frailty scale ranging from very fit to severely frail was established by Rockwood et al. and this correlated with outcomes of institutionalisation and survival [13]. These subjective assessments have limited generalisability and depend on the training, and geriatric experience and expertise of the observer.

### Frailty index

The FI developed by Rockwood and Mitnitski is based on the concept of frailty as an accumulation of deficits [10]. The quantity of deficits that accumulate over a person’s lifetime can be scored and higher numbers are associated with increasing likelihood of frailty. The deficits include defined symptoms, signs, diseases, disabilities and laboratory markers [10]. Deficits accumulate with age, are associated with adverse outcomes, occur across different domains, and can be measured over time [11].

FI figures correlate closely across countries, accumulate at a rate of approximately 3% per annum in the community, and are strongly correlated with mortality [14]. Ageing-associated adverse outcomes of decreased quality of life, increased hospitalisation and risk of death correlate more closely with the FI than with the phenotypic characterisation of frailty [15] or with chronological age [16].

## Pathophysiology of frailty

Frailty is associated with inflammation. It is unclear whether inflammation drives frailty, is a compensatory mechanism that occurs with frailty, or is an epiphenomenon [17]. A number of immunological and physiological impairments combine to increase the risk of frailty. Ongoing inflammation is a feature of HIV infection, despite plasma virological suppression with ART [18, 19]. A number of immunological and pathophysiological impairments that are found in ageing are also found in those with chronic HIV infection and combine to increase the risk of frailty [18, 19].

The immunological features of ageing and frailty consist of immunosenescence, immune activation and increased levels of circulating cytokines (C-reactive protein [CRP], tumour necrosis factor alpha [TNF-α], interleukin-6 [IL-6]) [20, 21]. Chronic cytomegalovirus (CMV) infection, which is common in PLHIV, may cause clonal expansion leading to immunosenescence, as well as causing immune activation and inflammation [22]. The role of chronic CMV in frailty has not been determined.

Physiological dysregulation associated with frailty was identified in the Women’s Health in Ageing Studies I and II (WHAS). Anaemia, inflammation, insulin-like growth factor-1 (IGF-1), dehydroepiandrosterone-sulphate (DHEA-S), haemoglobin A1c (HbA1c), micronutrients, adiposity and fine-motor speed were associated with a non-linear increase in frailty [23].

Mitochondrial loss from telomere shortening with ageing [24], and the effects of nucleoside analogue antiretrovirals used in the treatment of HIV [25], may also contribute to frailty by causing sarcopenia. Polypharmacy is also associated with a higher frailty risk in older people [26].

## Characteristics of and risk factors for frailty

Frail individuals often present with multiple and non-specific health complaints, fluctuating disability, falls and delirium, and are at higher risk for multiple adverse outcomes, including post-operative complications, poor responses to vaccination and functional decline. They tend to require longer hospital admissions, are more likely to require nursing home care, and are at greater risk of mortality [8].

Known risk factors for frailty include chronic kidney disease [27], cerebrovascular disease [28], cardiovascular disease [29] and smoking [30, 31], while increased exercise has a protective association with frailty [32]. Risk for frailty increases at both high and low BMI—those with BMI between 25 and 29.9 had the lowest FI scores and lowest prevalence of Fried frailty [33]. Social factors such as personal wealth and neighbourhood resources also impact frailty risk [34], and those with frailty in lower-income countries that have fewer resources have substantially higher rates of mortality [35].

HIV infection has also been associated with frailty using the Fried phenotype and FI [36, 37].

## Frailty in the HIV setting

Large, observational cohorts of HIV patients that measure ART use, clinical progress and development of co-morbidities have been useful in understanding associations with HIV infection, including the association between HIV and frailty.

The multicenter AIDS cohort study (MACS) defined frailty as three of: unplanned weight loss; exhaustion; low physical activity level; and slow walking speed. The study found that 4–10% of patients with HIV were frail, including up to 50% of patients over the age of 50 [5]. In the pre-ART era, there was a 10-fold increase in frailty in PLHIV compared to an HIV-negative population, but the prevalence of frailty in PLHIV halved with the introduction and use of ART [5]. In addition, low CD4+ T cell count and high HIV plasma RNA were associated with increased risk of frailty [5].

In the AGEhIV cohort study, PLHIV had a higher prevalence of frailty compared to HIV-negative individuals at all ages [6].

Frailty indexes specific to PLHIV have been established. The veterans ageing cohort study (VACS) developed a proxy FI that includes CD4+ T cell count, HIV viral load, hepatitis C co-infection, hepatic, renal and haematological markers, as well as d-dimer and soluble CD14, with the aim of predicting a mortality risk index in PLHIV [38]. The Modena HIV Metabolic Clinic developed a 37-item FI that includes HIV-related parameters to assess patients, and it has been found to be associated with multi-morbidity [39].

Higher rates of many co-morbidities that are associated with frailty occur in PLHIV compared to the general population, even adjusting for risk factors [40]. These co-morbidities include cardiovascular disease, metabolic disease, liver and kidney disease, osteoporosis and cancers. Frailty in PLHIV has been associated with increased falls, poorer functioning, decreased self-care, poor quality of life and depression, and neurocognitive impairment [41–44].

In addition to differences caused by constitution and other co-morbidities, there may also be differences in the risk of developing frailty between older patients with recently acquired HIV, patients with chronic HIV infection effectively suppressed on therapy, and patients with late diagnosis, untreated chronic HIV.

## Managing frailty in patients with HIV

Those treating HIV have become much more skilled in recognising and managing co-morbidity in PLHIV. There is now a need for further training so that HIV caregivers are equipped to recognise and manage ageing and frailty in this patient population as well.

Facilities for caring for those ageing with HIV, as well as those who have become frail, are currently quite variable in the levels of acceptance and comfort, and the ability to manage stigma and discrimination.

When managing frailty in PLHIV, physicians need to understand: when to intervene aggressively in the management of an older patient with a new HIV diagnosis to delay or prevent permanent debility and frailty; when to acknowledge that the patient has become frail; and the role of geriatric medicine in addressing the specific issues and needs of this patient such as maximising functional ability, preventing falls, reducing social isolation, and improving quality of life. The degree of frailty can fluctuate over time, which can complicate assessment of a patient.

There is a paucity of randomised clinical trial data on reversing frailty once it has been established. In the frailty interventions trial in elderly subjects (FITNESS) randomised controlled trial, simple measures such as randomised vitamin D supplementation and exercise over 10 weeks did not demonstrate improvements in self-reported physical health, physical performance, social activities or mental health [45].

A randomised multidisciplinary intervention over 12 months did significantly reduce frailty criteria and increased stability compared to controls, but did not impact on hospital admission, institutionalisation and death [46]. However, with 216 community participants, it was likely not powered to demonstrate these end points.

The comprehensive geriatric assessment (CGA) has been shown to be useful in managing elderly patients [47] and may be of benefit in patients with HIV with suspected frailty, even if they are below the usual geriatric age of 75 years.

There is variability in the evidence surrounding interventions that prevent frailty [48]. Epidemiological evidence suggests that interventions to assess and manage co-morbidities, reducing risk factors such as smoking, increasing exercise and optimising BMI, and improving personal and community resources, may reduce the risk of frailty [30–34].

In patients with HIV, managing frailty is likely to require early introduction of newer ART to maintain CD4+ T cell count, together with lifestyle interventions and proactive management, to reduce the risk of co-morbidities.

## Forum presentations

This report summarises presentations from the 2017 HIV innovation forum. Full speaker presentations can be accessed at http://www.innovationforum2017.com.au (password: HIVinnovation).

## Abbreviations

ART: antiretroviral therapy
CGA: comprehensive geriatric assessment
CMV: cytomegalovirus
CRP: C-reactive protein
DHEA-S: dehydroepiandrosterone-sulphate
FI: frailty index
FITNESS: frailty interventions trial in elderly subjects
HbA1c: haemoglobin A1c
IGF-1: insulin-like growth factor-1
IL-6: interleukin-6
MACS: multicenter AIDS cohort study
TNF-α: tumour necrosis factor alpha
VACS: veterans ageing cohort study
